# Safety outcomes and patients’ preferences for home-based intravenous enzyme replacement therapy (ERT) in pompe disease and mucopolysaccharidosis type I (MPS I) disorder: COVID-19 and beyond

**DOI:** 10.1186/s13023-023-02919-8

**Published:** 2023-10-27

**Authors:** Antonio Toscano, Olimpia Musumeci, Michele Sacchini, Sabrina Ravaglia, Gabriele Siciliano, Agata Fiumara, Elena Verrecchia, Melania Maione, Jennifer Gentile, Rita Fischetto, Grazia Crescimanno, Roberta Taurisano, Annalisa Sechi, Serena Gasperini, Vittoria Cianci, Lorenzo Maggi, Rossella Parini, Antonino Lupica, Maurizio Scarpa

**Affiliations:** 1 Full Professor of Neurology, ERN-NMD Center of Messina for Neuromuscular Disorders, Department of Clinical and Experimental Medicine, University of Messina, AOU Policlinico “G. Martino”, Via Consolare Valeria, 1, Messina, 98125 Italy; 2 Unit of Neurology and Neuromuscular Disorders, Department of Clinical and Experimental Medicine, University of Messina, Messina, 98125 ME Italy; 3 DH Hereditary metabolic-muscular diseases Meyer Hospital, Ground floor – DH Viale Pieraccini, 24, Florence, 50139 Italy; 4 IRCCS Fondazione Istituto Neurologico Nazionale C.Mondino, Via Mondino, 2, Pavia, 27100 PV Italy; 5 Department of Clinical and Experimental Medicine, S. Chiara Hospital - University of Pisa, Via Roma, 67, Pisa, 56126 Italy; 6 A.O.U. Policlinico - Pediatric Clinic and Regional Referral Center for Inherited Metabolic Diseases, Via Santa Sofia, 78, Catania, 95122 CT Italy; 7 Department of Aging, Neurological, Orthopedic and Head and Neck Sciences, Agostino Gemelli University Hospital Foundation, Via Giuseppe Moscati, 31, Rome, 00168 RM Italy; 8 Medical Manager Pompe Disease – Rare Diseases Specialty Care, Sanofi S.r.l., Viale Luigi Bodio 37/b, Milano, 20158 MI Italy; 9 Medical Manager Gaucher, MPS & ASMD, Sanofi S.r.l., Viale Luigi Bodio 37/b, Milano, 20158 MI Italy; 10 Policlinico di Bari Stabilimento Pediatrico Giovanni XXIII, Metabolic and Genetic Diseases, Piazza Giulio Cesare, 11, Bari, 70120 BA Italy; 11 Institute for Biomedical Research and Innovation (IRIB), National Research Council (CNR), Via La Malfa 153, Palermo, Italy; 12 Bambin Gesù Pediatric Hospital Piazza Sant’Onofrio, Rome (RM), 4 00165 Italy; 13 Regional Coordination Center for Rare Diseases, Udine University Hospital, Udine, 33100 UD Italy; 14 Unit of Inherited Metabolic Disorders, Pediatric Department, IRCCS San Gerardo Foundation dei Tintori, Via Pergolesi, 33 – Monza (MB), Tintori, Italy; 15 Great Metropolitan Hospital “Bianchi Melacrino Morelli” – Neurology, Reggio Calabria (RC), Via Melacrino, Calabria, 89100 Italy; 16 Neuroimmunology and Neuromuscular Diseases Unit, IRCCS Istituto Neurologico Besta, Via Celoria, 11, Milan, 20133 MI Italy; 17 ASST Monza - Rare Disease Center, San Gerardo hospital in Monza, Via Pergolesi, Monza, 33 - 20900 MB Italy; 18 AOU Policlinico P. Giaccone of Palermo, Via del Vespro 129, Palermo, 90127 Italy; 19 Regional Coordinating Center for Rare Diseases, Udine University Hospital, Udine, 33100 Italy

**Keywords:** Enzyme replacement therapy, Pompe disease, Home therapy, Mucopolysaccharidosis, Treatment adherence, Safety

## Abstract

**Background:**

The Italian Medicines Agency (AIFA) demands precise information on benefit/risk profile of home-based enzyme replacement therapy (ERT) for the treatment of patients with Pompe disease and Mucopolysaccharidosis type I (MPS I). This passage is necessary to obtain the authorization for ERT home therapy, even after the coronavirus disease-19 (COVID-19) pandemic period. This research intends to evaluate the safety, treatment satisfaction, and compliance of MPS I patients treated with laronidase (Aldurazyme®) and Pompe Disease patients treated with alglucosidase alfa (Myozyme®) in a homecare setting.

**Results:**

We report herein an early interim analysis of the HomERT (Home infusions of ERT) study, a multicenter, non-interventional, double-cohort study that retrospectively analyzed 38 patients from 14 sites in Italy: cohort A (Pompe disease − 32 patients) and cohort B (MPS I − 6 patients). Among the selected patients who started home therapy before enrollment, the average number of missed home-based infusions was 0.7 (1.3) in cohort A and 3.8 (6.4) in cohort B with no return to the hospital setting. Irrespective of the treatment location, 3 prior ADRs per cohort were reported. The majority of patients preferred home-based infusions (cohort A: 96.9%; cohort B: 100%): the main reason was attributed to treatment convenience (cohort A: 81.3%; cohort B: 83.3%). Despite the underlying conditions, most patients self-evaluated their health as “good” (cohort A: 50%; cohort B: 83.3%).

**Conclusions:**

Evidence of favorable safety profile, improved treatment compliance and personal satisfaction validates the use of ERT with laronidase and alglucosidase alfa as a strong candidate for home therapy.

## Introduction

Lysosomal storage diseases (LSDs) are a group of at least 41 genetically distinctive [1] inherited rare metabolic disorders with overall incidence estimated at approximately 1 in 4,000 to 1 in 8,000 live births [1–6]. Each disorder is due to an individual enzyme deficiency [7], with Pompe disease and mucopolysaccharidoses (MPS) caused by deficiency of α-glucosidase and α-L-iduronidase, respectively. This leads to pathological accumulation of various substrates within the lysosomes, which causes a broad spectrum of clinical manifestations leading often to multiorgan damage [8, 9].

Early diagnosis is essential to limit the irreversible organ damage associated with clinical progression. Extensive and real-world clinical studies have confirmed the efficacy and safety of enzyme replacement therapy (ERT) like alglucosidase alfa (Myozyme®) and laronidase (Aldurazyme®) to respectively treat Pompe disease and MPS. Although disease progression can be observed in both Pompe disease and MPS in patients with good treatment adherence, it tends to be much more rapid in patients without treatment or with poor treatment compliance [10–12]. Case reports of rapid cessation of ERT therapy suggested that prolonged interruptions cause not only a loss of the beneficial effects of therapy but also a significant worsening of clinical status [13–15]. These reports underline the importance of maintaining an appropriate treatment compliance.

ERT is often administered in a hospital setting due to safety concerns. However, hospital-based therapy can be stressful and inconvenient over time causing a negative impact on quality of life (QoL) [16, 17]. Patients and their families feel that home-based therapy is less stressful and more comfortable than prolonged hospital-based treatment [18, 19].

The advent of the Coronavirus Disease-19 (COVID-19) pandemic imposed an unprecedented challenge on LSD patients for receiving ERT at hospitals and maintaining adherence. An Italian study assessing the impact of COVID-19-related healthcare crisis on LSD treatment demonstrated that patients were in favor of changing from hospital to home therapy, not only during the pandemic, but even after. Except for 1 patient who missed 1 injection, all patients on home treatment received their infusions without interruptions. Among patients on hospital-based ERT, 49% experienced treatment disruptions [20]. Thus, home therapy seemed to be the most efficient strategy to sustain therapeutic access during the outbreak.

Thus, the Agenzia Italiana del Farmaco (AIFA) temporarily and exceptionally, during the pandemic, authorized (341/2020) the home infusion in Italy for Pompe disease and MPS I patients who had already undergone hospital-based ERT for a certain period with no incidence of adverse events. To obtain permanent authorization even after the pandemic, AIFA demands continuous risk/benefit balance evaluation of home therapy.

The HomERT (Home infusions of ERT) study aims to obtain safety information, infusion compliance and treatment satisfaction on home therapy of Pompe disease patients with Myozyme® (alglucosidase alfa) and of MPS I patients with Aldurazyme® (laronidase) in a real-world setting in Italy.

## Methods

### Study Design

#### Interim analysis

This article describes the findings of an interim analysis from the HomERT study. The interim analysis was conducted at the end of the enrollment period of the HomERT study program to have a first insight of the safety, infusion compliance and treatment satisfaction of ERT in a homecare setting. Data on patient reports, infusion characteristics and Adverse Drug Reactions (ADRs) from Italian patients with Pompe disease and MPS I who started ERT infusions were collected from 14th October 2021 to 31st August 2022 and retrospectively analyzed. The observation started from the first ERT infusions in a homecare setting until the enrollment period. Treatment satisfaction questionnaire was administered at the enrollment visit by the Investigators. Only visits performed by the cut-off date (i.e., August 31st, 2022) were reported and analyzed.

### HomERT study program

The HomERT study is an Italian, multicenter, non-interventional, double-cohort study with both retrospective and prospective data collection to obtain information related to safety and patient satisfaction of treatment of Pompe disease and MPS I with ERT in a homecare setting. The HomERT study program is still ongoing for prospective observation that will last for 12 months from enrollment. There will be no imposed protocol visits or procedures. During the control visits conducted every 6–12 months, the Investigators will administer the questionnaires again to collect information on patient-reported outcomes and will record any documented clinical data occurring during the home infusions.

The study protocol and the questionnaire were approved by the local ethics committees of all participating sites. This study was conducted in accordance with the Declaration of Helsinki, and Good Pharmacoepidemiology Practices (GPPs). Informed consent was obtained from each participant before enrollment in the study. Eligibility of the participants were suggested by the physicians, usually treating patients at the hospital, and based on standard treatment options as determined by Italian regulations (including AIFA authorization 341/2020 for Pompe disease patients and MPS I patients). Dosage and dosage regimen were in accordance with the Summary of Product Characteristics (SmPC).

### Study population

The study population included 38 patients from 14 sites in Italy. The study population was categorized into 2 cohorts [cohort A (Pompe disease): n = 32 patients; cohort B (MPS I): n = 6 patients]. Inclusion criteria were (A) For cohort A: Pompe disease patients with confirmed acid alpha-glucosidase (GAA) enzyme deficiency receiving Myozyme® (alglucosidase alfa) in a homecare infusion setting, according to authorized clinical practice and the approved risk management plan document; and for cohort B: MPS I patients with confirmed deficiency of alpha-L-iduronidase receiving Aldurazyme® (laronidase) in a homecare infusion setting according to authorized clinical practice and the approved risk management plan document; (B) Written informed consent before enrollment.

Also, for both cohorts the study included patients already in a home infusion setting prior to enrollment (or) patients selected for transfer to a home infusion setting at enrollment.

### Questionnaire

A patient satisfaction questionnaire, designed “ad hoc”, was used to assess patient satisfaction related to the home infusions, including potential benefits in terms of stress, time, health, and QoL. This questionnaire was specifically developed for this kind of survey. Satisfaction questionnaires were administered by the Investigators to the patients at the enrollment visit, at each control site-visit and at the end of the study/discontinuation visit. In this interim analysis, questionnaires were completed by the patient/caregiver, as applicable, at the enrollment visit. Patients completing the questionnaire had received prior ERT home-based infusions.

### Statistical methods

As the descriptive aim of the study is related to the observation of patients with rare diseases, sample size was estimated based on the feasibility to enroll patients treated in a homecare setting. Approximately 44 patients for cohort A and 16 patients for cohort B were estimated for this study. Descriptive statistics (n, mean, median, standard deviation (SD), range, min, max for continuous variables, and count and percentage for categorical variables) were used to summarize treatment exposure, safety outcomes and patient’s characteristics. Data were analyzed and presented by cohorts (cohort A and cohort B).

The primary analysis evaluated the incidence of ADRs based on seriousness and intensity. The secondary analysis evaluated patient satisfaction assessed by means of a patient satisfaction questionnaire and treatment compliance was assessed as the number of missed infusions vs. planned and/or return to the hospital setting (with reasons). Analyses were performed on data of baseline demographics, including medical history based on the Medical Dictionary for Regulatory Activities (MedDRA). Results of vital signs and laboratory parameters were categorized as low/normal/high based on clinical normal ranges, and abnormal values were flagged. Also, abnormalities on physical examination were flagged. Statistical analyses were performed by means of SAS® release 9.4 (SAS Institute, Inc., Cary, NC, USA). Data from all sites were pooled and summarized.

## Results

### Respondents’ disposition, socio-demographic and clinical characteristics

Screened cases were patients who provided a written Informed Consent Form (ICF) to participate in the study or for whom written ICF was obtained from parent(s)/legal guardian by the cut-off date, i.e., August 31st, 2022. A total of 38 patients were screened in the study. All of them passed screening (100%) and were enrolled in 2 cohorts. Of these, 32 patients were categorized into cohort A (Pompe disease) and 6 patients were categorized into cohort B (MPS I). An overview of patients’ demographics and baseline characteristics is illustrated in Table 1.

**Table 1:** Baseline Patient Demographics and Disease Characteristics

	*Cohort A* *(N = 32)*	*Cohort B* *(N = 6)*	*Total* *(N = 38)*
**Demographics**			
Age (years)			
Mean (SD)	45.9 (24.50)	23.2 (13.72)	42.3 (24.48)
Q1; Q3	22.5; 66.5	14.0; 37.0	19.0; 65.0
Range	3; 78	9; 43	3; 78
Age, n (%)			
0–18 years	6 (18.75)	3 (50.00)	9 (23.68)
19–35 years	7 (21.88)	1 (16.67)	8 (21.05)
36–55 years	3 (9.38)	2 (33.33)	5 (13.16)
56–70 years	11 (34.38)	0	11 (28.95)
over 70 years	5 (15.63)	0	5 (13.16)
Sex, n (%)			
Female	16 (50.00)	3 (50.00)	19 (50.00)
Male	16 (50.00)	3 (50.00)	19 (50.00)
**Disease Characteristics**			
Diagnosis, n (%)			
MPS I disease			
Hurler – Scheie	0	3 (50.00)	3 (7.89)
Scheie for MPS I	0	3 (50.00)	3 (7.89)
Pompe disease			
Infantile-onset Pompe Disease	5 (15.63)	0	5 (13.16)
Late-onset Pompe Disease	27 (84.38)	0	27 (71.05)
Cognitive delay, n (%)			
No	32 (100.00)	4 (66.67)	36 (94.74)
Yes	0	2 (33.33)	2 (5.26)
Age at diagnosis (years)			
Mean (SD)	31.3 (23.53)	10.0 (9.06)	28.0 (23.18)
Q1; Q3	4.5; 52.0	5.0; 10.0	5.0; 50.0
Range	0; 64	4; 28	0; 64
Age at start of ERT administration in hospital setting (years)			
Mean (SD)	35.4 (25.16)	12.7 (9.79)	31.8 (24.77)
Q1; Q3	9.5; 58.5	6.0; 20.0	7.0; 54.0
Range	0; 66	4; 29	0; 66
Time from diagnosis to start of ERT administration in hospital setting (months)			
Mean (SD)	50.5 (61.44)	31.0 (60.29)	47.4 (60.88)
Q1; Q3	3.5; 100.5	5.0; 10.0	4.0; 99.0
Range	0; 211	4; 154	0; 211
Patients Starting The ERT Administration In A Homecare Setting, N (%) *	**30 (93.75)**	**6 (100.00)**	**36 (94.74)**
Reasons for switching to homecare setting, n (%) *			
COVID-19 related	19 (63.33)	1 (16.67)	20 (55.56)
Patient request	8 (26.67)	3 (50.00)	11 (30.56)
Center distant	3 (10.00)	0	3 (8.33)
Other	0	2 (33.33)	2 (5.56)
Age at start of ERT administration in a homecare setting (years)*			
Mean (SD)	47.9 (22.87)	17.7 (15.64)	42.8 (24.46)
Q1; Q3	26.0; 67.0	7.0; 34.0	19.5; 64.5
Range	8; 78	5; 41	5; 78
Time from diagnosis to start of ERT administration in homecare setting (months)*			
Mean (SD)	173.2 (85.79)	93.7 (126.51)	159.9 (96.37)
Q1; Q3	102.0; 248.0	15.0; 157.0	83.0; 238.5
Range	12; 315	15; 326	12; 326
Time to switch to homecare setting (months)*			
Mean (SD)	119.2 (52.76)	62.5 (75.78)	109.8 (59.89)
Q1; Q3	82.0; 168.0	11.0; 147.0	58.5; 163.5
Range	10; 200	8; 172	8; 200
Time from start of ERT administration in homecare setting to screening (months)*			
Mean (SD)	14.6 (7.51)	69.5 (40.41)	23.8 (26.66)
Q1; Q3	9.0; 19.0	27.0; 106.0	10.0; 23.0
Range	0; 26	26; 116	0; 116
Patients who started the ERT administration in homecare setting prior to enrollment, n (%)*			
No	3 (9.38)	0	3 (7.89)
Yes	29 (90.63)	6 (100.00)	35 (92.11)
**Other Medical Conditions**			
Patients with prior medical conditions, n (%)	16 (50.00)	6 (100.00)	22 (57.89)
Patients with ongoing medical conditions, n (%)	26 (81.25)	5 (83.33)	31 (81.58)
Patients reporting the presence of any significant respiratory disease, n (%)	18 (56.25)	1 (16.67)	19 (50.00)
Patients with evidence of serious obstructive airway disease, n (%) ^$^			
Other	10 (55.56)	1 (100.00)	11 (57.89)
Respiratory Failure	8 (44.44)	0	8 (42.11)
Predicted forced vital capacity, (%) ^$^			
n	18	1	19
Mean (SD)	50.4 (20.07)	38.0	49.8 (19.71)
Q1; Q3	36.0; 68.0	38.0; 38.0	36.0; 68.0
Range	10; 82	38; 38	10; 82

*Q1 = 1st quartile; Q3 = 3rd quartile; SD = Standard Deviation.*

*Percentages were computed on patients belonging to the Enrolled population within each considered group.*

* Computed only for patients starting the homecare setting administration.

^*$*^ *Computed only for patients reporting the presence of any significant respiratory disease.*

*Cohort A is consisting of Pompe disease patients receiving Myozyme in a homecare setting, while cohort B is composed of MPS I patients receiving Aldurazyme in a homecare setting.*

***Age at start date of ERT administration in hospital setting (years)*** *is calculated as: age at diagnosis + (start date of ERT in hospital setting – date of diagnosis)/365.25.*

***Time from diagnosis to start of ERT administration in hospital setting (months)*** *is calculated as: (start date of ERT administration in hospital setting - date of diagnosis)/30.4375.*

***Age at start of ERT administration in a homecare setting (years)*** *is calculated as: age at diagnosis + (start date of ERT in homecare setting – date of diagnosis)/365.25.*

***Time from diagnosis to start of ERT administration in homecare setting (months)*** *is calculated as: (start date of ERT administration in homecare setting - date of diagnosis)/30.4375.*

***Time to switch to homecare setting (months)*** *is calculated as: (start date of ERT administration in homecare setting – start date of ERT administration in hospital setting)/30.4375.*

***Time from start of ERT administration in homecare setting to screening (months)*** *is calculated as: (Screening visit - start date of ERT administration in homecare setting)/30.4375.*

The mean (SD) age was 45.9 (24.5) years in cohort A and 23.2 (13.7) years in cohort B; in both cohorts, males and females were each 50%. The more frequent age class was 56–70 years (11 patients, 34.4%) in cohort A, and 0–18 years (3 patients, 50%) in cohort B. In cohort A, 84.4% of patients reported Late-onset Pompe Disease (LOPD), with no cognitive delay. In cohort B, 50% patients each reported Hurler – Scheie and Scheie, and 33.3% patients with cognitive delay. The mean (SD) age at diagnosis was 31.3 (23.5) years in cohort A and 10 (9.1) years in cohort B. Mean (SD) age at start of ERT administration in a hospital setting was 35.4 (25.2) years in cohort A and 12.7 (9.8) years in cohort B; and mean (SD) time from diagnosis to start of ERT administration in a hospital setting was 50.5 (61.4) months in cohort A and 31.0 (60.3) months in cohort B.

All the patients belonging to cohort B (6 patients, 100%) and a majority of cohort A (29 patients, 90.6%) started ERT at home even before enrollment in the study, while three patients started ERT at the hospital initially and were later shifted to home therapy. Mean age (SD) at start of ERT administration in a home setting was 47.9 (22.9) years in cohort A and 17.7 (15.6) years in cohort B; and mean (SD) time from diagnosis to start of ERT administration in a home setting was 173.2 (85.8) months in cohort A and 93.7 (126.5) months in cohort B.

Main reasons for switching to home therapy in cohort A was COVID-related (63.3%), patient request (26.7%) and center distance (10%); and in cohort B was patient request (50%), other reasons (33.3%) (improvement in the subject’s stature and medical considerations) and COVID-related (16.7%). Mean time to switch to a homecare setting was 119.2 (52.8) months in cohort A and 62.5 (75.78) months in cohort B. The mean time from start of ERT administration in a homecare setting to screening was 14.60 (7.5) months for cohort A and 69.50 (40.4) months for cohort B.

### Preferences for Home Therapy based on Safety Outcomes and Tolerability

Among the enrolled population who started the homecare setting before enrollment, only 2 patients/cohort (cohort A: 6.9%; cohort B: 33.3%) reported at least one prior ADR. Irrespective of hospital- or home-based setting, the proportion of patients with ADRs was low in both cohorts reflecting highly favorable tolerability.

However, among the 3 prior ADRs reported per cohort, all 3 ADRs (100%) in cohort A and 1 out of 3 ADRs (33.3%) in cohort B occurred in a hospital setting. One patient (3.5%) each in both cohorts reported ADRs associated with hospital-based infusions limited to non-serious mild “Erythema and itching”, serious moderate “Urticaria” and serious moderate “Dyspnea” in cohort A; and non-serious mild “Rash” in cohort B. ADRs associated with home-based infusions were not reported in any of the patients in cohort A and 1 patient in cohort B reported 2 events of non-serious mild “Pyrexia”. **(**Table 2**)**

**Table 2:** Summary of Prior Home Infusion in Enrolled Patients Who Started the Homecare Setting Before Enrollment

	Cohort A (N = 29)	Cohort B (N = 6)	Total (N = 35)
Number of dilutions performed by patients			
Mean (SD)	1.0 (0.00)	1.0 (0.00)	1.0 (0.00)
Q1; Q3	1.0; 1.0	1.0; 1.0	1.0; 1.0
Range	1; 1	1; 1	1; 1
**Any change in dilution infusion, n (%)**			
Yes	0	0	0
No	29 (100.00)	6 (100.00)	35 (100.00)
**Prior home infusion by rate of administration, n (%)**			
Every 2 weeks	27 (93.10)	0	27 (77.14)
Weekly	2 (6.90)	6 (100.00)	8 (22.86)
**Infusions by administration of any pre-medication, n (%)**			
No	22 (75.86)	3 (50.00)	25 (71.43)
Yes	7 (24.14)	3 (50.00)	10 (28.57)
**Number of missed ERT infusions during ERT administration in a homecare setting**			
Mean (SD)	0.7 (1.26)	3.8 (6.40)	1.2 (2.96)
Q1; Q3	0.0; 1.0	0.0; 6.0	0.0; 1.0
Range	0; 4	0; 16	0; 16
**Average duration of infusion (hours)** ^**a**^			
Mean (SD)	4.7 (1.16)	3.7 (0.52)	4.5 (1.15)
Q1; Q3	4.0; 6.0	3.0; 4.0	4.0; 5.0
Range	3; 7	3; 4	3; 7
**Prior Adverse Drug Reactions (ADRs) n (%)** ^**b**^			
Number of prior ADRs	3	3	6
Number of prior ADRs by setting			
Home infusion	0	2 (66.67)	2 (33.33)
Hospital infusion	3 (100.00)	1 (33.33)	4 (66.67)
Serious prior ADRs			
No	1 (33.33)	3 (100.00)	4 (66.67)
Yes	2 (66.67)	0	2 (33.33)
Intensity of prior ADRs			
Mild	1 (33.33)	3 (100.00)	4 (66.67)
Moderate	2 (66.67)	0	2 (33.33)
*MedDRA System organ class/* Preferred term ^c^			
*General disorders and administration site conditions*	0	1 (16.67)	1 (2.86)
Pyrexia	0	1 (16.67)	1 (2.86)
*Immune system disorders*	1 (3.45)	0	1 (2.86)
Urticaria	1 (3.45)	0	1 (2.86)
*Respiratory, thoracic, and mediastinal disorders*	1 (3.45)	0	1 (2.86)
Dyspnea	1 (3.45)	0	1 (2.86)
*Skin and subcutaneous tissue disorders*	1 (3.45)	1 (16.67)	2 (5.71)

*ERT = Enzyme Replacement Therapy; ADR = Adverse Drug Reaction, MedDRA = Medical Dictionary for Regulatory Activities; Q1 = 1st quartile; Q3 = 3rd quartile; SD = Standard Deviation*

*Percentages were computed on patients belonging to the enrolled population who started the homecare setting before enrollment within each considered group; cohort A is consisting of Pompe disease patients receiving Myozyme in a homecare setting, while cohort B is composed of MPS I patients receiving Aldurazyme in a homecare setting.*

^*a*^ *Average duration of infusion was the time spent performing home infusions (hours) reported by patients for each performed dilution*

^*b*^ *Computed on the total number of prior ADRs occurred within each considered group; Each subject could have more than one prior Adverse Drug Reaction, but they are counted only once for each condition/row.*

^*c*^ *Terms were coded using MedDRA, version 24.0.*

### Influence of treatment compliance and treatment satisfaction on patients’ Preferences of Home Therapy

#### Treatment compliance

Among enrolled patients who started the homecare setting before enrollment, favorable treatment adherence to ERT infusions was observed. **(**Table 2**)** The number of missed ERT infusions in a homecare setting was very low [0.7 (1.3) in cohort A and 3.8 (6.4) in cohort B]. After receiving ERT at home, there were no cases of patients who returned to receiving ERT administration at hospital.

The majority of the patients in cohort A (93.1%) were administered infusions for 4.7 (1.2) hours biweekly while weekly administration of 3.7 (0.5) hours was performed in cohort B (100%). About 24.1% in cohort A and 50% in cohort B underwent infusions following pre-medication administration.

In both cohorts, about one-fourth of the population (cohort A: 21.9%; cohort B: 33.3%) had venous access at home. In cohort A, “peripheral intravenous” (42.9%) and “totally implanted venous device” (42.9%) were the most commonly used venous devices. In cohort B, all the patients who had venous access used a “peripherally inserted central catheter” (100%).

### Treatment satisfaction at Screening

All patients (100%) completed the home infusion satisfaction questionnaire at screening. Nearly all the patients (cohort A: 96.9%; cohort B: 100%) preferred to receive ERT infusions at home. Treatment preference to homecare therapy was attributed to more treatment convenience (cohort A: 81.3%; cohort B: 83.3%), less transportation requirement (cohort A: 65.6%; cohort B: 50%), perception of less stressfulness (cohort A: 62.5%; cohort B: 50%), and less impact on daily activities (cohort A: 43.8%; cohort B: 33.3%).

Despite the underlying condition (LSD), a higher proportion of patients self-evaluated their health as “good” (cohort A: 50%; cohort B: 83.3%). Of significance, 1 patient (16.7%) in cohort B self-assessed his health as “very good”, while in cohort A more variability was observed: 2 patients (6.3%) judged their health to be “excellent”, 5 patients (15.6%) as “very good health”, 4 patients (12.5%) responded “fair health”, and the remaining 5 patients (15.6%) responded “poor health”.

Clinical outcomes based on responses varied by cohort when comparing the improvement observed in physical health after receiving ERT at home versus at the hospital. In cohort A, 19 (59.4%) patients rated their physical health as “about the same”, while 6 patients (18.75%) rated it as “much better now” or “somewhat better now”. In cohort B, 3 patients (50%) rated their physical health as “much better now”, 2 patients (33.33%) as “somewhat better now”, and 1 patient (16.67%) as “about the same”. **(**Table 3**)**

**Table 3:** Impact of the home-based infusions on Treatment Satisfaction in Enrolled population

	*Cohort A* *(N = 32)* *n (%)*	*Cohort B* *(N = 6)* *n (%)*	*Total* *(N = 38)* *n (%)*
Number of patients who completed the home infusion satisfaction questionnaire at screening	32 (100.00)	6 (100.00)	38 (100.00)
Where do you prefer to take your ERT infusions?			
Hospital/clinic near your home	1 (3.13)	0	1 (2.63)
Home	31 (96.88)	6 (100.00)	37 (97.37)
Why do you prefer to take your ERT infusion in home setting? ^a^			
More convenient	26 (81.25)	5 (83.33)	31 (81.58)
Less stressful	20 (62.50)	3 (50.00)	23 (60.53)
Daily activities are less disrupted	14 (43.75)	2 (33.33)	16 (42.11)
Work/school are less disrupted	8 (25.00)	3 (50.00)	11 (28.95)
Family life is less disrupted	8 (25.00)	3 (50.00)	11 (28.95)
Less transportation needed (drive, take bus/taxi/train)	21 (65.63)	3 (50.00)	24 (63.16)
More clinical supervision	3 (9.38)	1 (16.67)	4 (10.53)
Feel less socially isolated	1 (3.13)	1 (16.67)	2 (5.26)
Other	2 (6.25)	0	2 (5.26)
In general, and despite your LSD, would you say your health is:			
Excellent	2 (6.25)	0	2 (5.26)
Very good	5 (15.63)	1 (16.67)	6 (15.79)
Good	16 (50.00)	5 (83.33)	21 (55.26)
Fair	4 (12.50)	0	4 (10.53)
Poor	5 (15.63)	0	5 (13.16)
If you are receiving ERT at home, how would you rate health in general compared to the period you received ERT in the hospital?			
Much better now	6 (18.75)	3 (50.00)	9 (23.68)
Somewhat better now	6 (18.75)	2 (33.33)	8 (21.05)
About the same	19 (59.38)	1 (16.67)	20 (52.63)
Missing	1 (3.13)	0	1 (2.63)

*ERT = Enzyme Replacement Therapy, LSD = Lysosomal Storage Disease.*

*Percentages were computed on patients belonging to the enrolled population within each considered group.*

*Cohort A is consisting of Pompe disease patients receiving Myozyme in a homecare setting, while cohort B is composed of MPS I patients receiving Aldurazyme in a homecare setting.*

^*a*^ *More answers were allowed*.

## Discussion

The results of this retrospective interim analysis of the HomERT study program provide healthcare policy makers and physicians with a new important dimension in the understanding of safety, treatment outcomes and treatment satisfaction of homecare therapy of Pompe disease and MPS I in Italy. Unprecedented challenges imposed by COVID-19 have urged the need to provide home-based infusions for LSD patients in order to avoid frequent travels and hospital admissions to receive periodical IV infusions of ERT.

Owing to safety concerns, home therapy of ERT infusions is not authorized in many countries. In this study, ~ 90% of the enrolled population started ERT administration in a homecare setting prior to enrollment and were on home therapy for 14.6 (7.5) months in cohort A and 69.5 (40.4) months in cohort B. In both groups, laboratory parameters (not shown), vital signs (not shown) and physical examination (not shown) were within the limits of normal clinical range with minimal clinically relevant anomalies, indicating that patients were stable at screening, irrespective of the treatment location (home vs. hospital). Safety data presented from the Italian cohorts exhibited uneventful infusions in a hospital setting with acceptable tolerability profile recommending the study patients as ideal candidates for complete transitioning to home-based treatment. Overall, as opposed to hospital-based infusion, the incidence of prior ADRs was lower on homecare infusions with good safety profile. Seriousness criteria and intensity of ADRs are the key factors that should be considered for safety. ADRs occurring in a homecare setting were not reported in any of the patients in cohort A and only 1 patient in cohort B, limited to 2 episodes of non-serious, mild “Fever”. None of the reported ADRs warranted urgent medical intervention. The current findings are consistent with previous experiments on home-based infusion of ERT in MPS I patients [21] and Pompe disease [22], indicating that the rate of ADRs during home therapy was quite low with mild or moderate intensity and can be readily managed without treatment interruption. Precisely, the data from this interim analysis suggest that in patients with Pompe disease and MPS I, home-based ERT infusions are equivalently safe as hospital-based ERT infusions.

These results were also confirmed by a recent study conducted on 18,380 infusions with alglucosidase alfa in 121 adult patients; 4961 infusions (27.0%) were given in hospital and 13,419 (73.0%) were given at home. Infusion-associated reactions (IARs) occurred in 144 (2.9%) hospital infusions and 113 (0.8%) home infusions; 115 (79.9% of 144) IARs in hospital and 104 (92.0% of 113) IARs at home were mild, 25 IARs (17.4%) in hospital and 8 IARs (7.1%) at home were moderate, and very few severe IARs occurred (4 IARs in hospital [2.8%] and 1 IAR at home [0.9%]). Only one IAR in the home situation required immediate clinical evaluation in the hospital. The study concluded that alglucosidase alfa can be administered safely in the home situation, provided the appropriate infrastructure is present [23].

Home therapy experience abroad [24] and in pandemic conditions [20] has shown that ERT infusions at home improve QoL by affording patients independence and control of the disease, and reduce hospital resource usage. In this study, transition to home infusion therapy was due to COVID-19, traveling reasons, upon patients’ request and other reasons (i.e., For improvement in the subject’s stature and medical considerations). Approximately 25% of patients had peripheral venous access or a totally implanted venous access device in situ at home. This study speculated that the majority of patients with Pompe disease and MPS I were more comfortable with a home-based infusion setting as the patients were able to perform an IV dilution independently with no change in the concentration of the dilution throughout the treatment period and adhered to the recommended dosage regimen of alglucosidase alfa [25] and laronidase [26]. In the present study, treatment compliance was measured by the number of missed ERT infusions as defined by Linthorst et al. [27] and the home infusion program positively influenced infusion compliance by reducing the number of therapy interruptions. Apparently, no instances of relapse into hospital infusion settings were observed. Having the derived results as evidence, we can argue that improved ERT compliance will inevitably have a positive impact on treatment outcomes over the long term.

Since patients may have to travel considerable distance to some hospitals, regular visits may be stressful, time-consuming, tiring and, sometimes, economically burdensome. Of significance, enrolled patients in this analysis had to spend time only for home-based infusions as there were no travel requirements. This is interesting, as data from a previous report indicated that hospital-based infusions, in addition to the infusion time, required an average of more than 2 h to travel and also the support of a caregiver to travel to the hospital [28].

Irrespective of the cohorts, ~ 95% of patients claimed that home-based therapy increased their overall treatment satisfaction in terms of level of comfort, treatment convenience, flexibility, cancellation of travel costs and QoL while reducing their stress and, likewise, requirement of transportation. This result is in line with earlier published work, reporting that administering ERT IV infusions at home was more convenient, effective, less stressful, and had a lower impact on family life [18]. The observation of the current analysis differs from the outcomes of an Italian regional survey that elaborated that hospital ERT infusions were less stressful (40% of patients) and safer (93%) owing to the immense medical support and close monitoring from medical professionals. However, this preference towards hospital-based infusions is attributed to the enrollment of severe cases and the patients’ caregivers had no professional obligations as they were retired. [16].

With the HomERT observational study, the patients’ perception of health was used as QoL indicator. Despite the LSD manifestations, the current cohort possessed an optimistic attitude about their health on ERT treatment administered in a homecare setting. The majority of respondents conveyed that their health on home therapy greatly improved or was comparable to the clinical effect of hospital care. Notably, a previous publication recommended that a successful home treatment program include a selection of stable patients at the discretion of a physician, equipped homecare nursing, education of patients’ caretakers, and outlining management strategy for anticipated anaphylaxis and ADRs [29].

One of the strengths of this study is that all recruited participants were enrolled from Italy, minimizing ethnic and cultural bias that may confound patient preference. The advantage of using data obtained from non-interventional studies implies obtaining data in a normal clinical setting under real-life conditions, which are more representative of the study population of interest and the clinical outcomes under observation. Despite the aforementioned strengths, there are potential limitations worth discussion. The main limitations of this interim analysis are attributed to its observational retrospective nature that may involve patient selection bias, incomplete or missing data and lack of internal validity (no control group), difficulty in interpreting or verifying documented information, and variability between patients in the quality of documentation. Furthermore, treatment satisfaction assessed through patient responses are subjective and may involve a risk of recall bias, hence some outcomes might not be accurate from a medical perspective. Enrolled patients were already in a home infusion setting prior to enrollment, and the preference for home therapy over hospital therapy by experienced person may differ from the judgment of a participant new to treatment. Lack of in-depth investigations that encompasses bigger cohort size or compare the attitudes between young and adult patients could have added a new opinion to treatment preference.

The authors foresee conditioned regulatory approval of home therapy for LSDs as the main hinderance to establish home-based infusion of ERT in Italy. The authors advocate the Italian healthcare policy makers to take into consideration the positive outcomes gained from this interim analysis of the still ongoing HomERT program for further optimization of the protocol with ERT home-based infusions in Pompe disease and MPS I.

## Conclusion

In summary, a large number of patients with Pompe disease and MPS I contemplated home-based therapy to be more convenient, more flexible, and less stressful than hospital-based therapy. The burden of hospital therapy on work and family life was minimized by seamlessly integrating infusions into the patients’ daily routine through home therapy. Based on our real-world data retrospective interim analysis of the HomERT program, we found no evidence that the safety of laronidase and alglucosidase alfa substantially change with home infusion. This proved that for Pompe disease and MPS I, home-based ERT infusion is equally safe as hospital-based infusion, feasible and might alleviate the burden of life-long intravenous treatment in these patients.

## Data Availability

The data that support the findings of this study are not openly available due to reasons of sensitivity and are available from the corresponding author upon reasonable request. Data are located in controlled access data storage at Sanofi s.r.l. Sole Shareholder.
